# Inpatient Cognitive Behaviour Therapy for Anorexia Nervosa: A Randomized Controlled Trial

**DOI:** 10.1159/000350058

**Published:** 2013-09-20

**Authors:** Riccardo Dalle Grave, Simona Calugi, Maddalena Conti, Helen Doll, Christopher G. Fairburn

**Affiliations:** ^a^Department of Eating and Weight Disorders, Villa Garda Hospital, Garda (Vr), Italy; ^b^Department of Health Sciences, Leicester University, Leicester, Oxford, UK; ^c^Department of Psychiatry, Warneford Hospital, Oxford University, Oxford, UK

**Keywords:** Anorexia nervosa, Body mass index, Cognitive behaviour therapy, Eating disorders, diagnosis, therapy, Female, Follow-up studies, Humans, Inpatient treatment, Relapse

## Abstract

**Background:**

The aim of this study was to compare the immediate and longer-term effects of two cognitive behaviour therapy programmes for hospitalized patients with anorexia nervosa, one focused exclusively on the patients' eating disorder features and the other focused also on mood intolerance, clinical perfectionism, core low self-esteem or interpersonal difficulties. Both programmes were derived from enhanced cognitive behaviour therapy (CBT-E) for eating disorders.

**Methods:**

Eighty consecutive patients with severe anorexia nervosa were randomized to the two inpatient CBT-E programmes, both of which involved 20 weeks of treatment (13 weeks as an inpatient and 7 as a day patient). The patients were then followed up over 12 months. The assessments were made blind to treatment condition.

**Results:**

Eighty-one percent of the eligible patients accepted inpatient CBT-E, of whom 90% completed the 20 weeks of treatment. The patients in both programmes showed significant improvements in weight, eating disorder and general psychopathology. Deterioration after discharge did occur but it was not marked and it was restricted to the first 6 months. There were no statistically significant differences between the effects of the two programmes.

**Conclusions:**

These findings suggest that both versions of inpatient CBT-E are well accepted by these severely ill patients and might be a viable and promising treatment for severe anorexia nervosa. There appears to be no benefit from using the more complex form of the treatment.

## Introduction

The mainstay of the treatment of eating disorders is outpatient treatment. It is less disruptive and costly than inpatient or day patient treatment, which are not necessary in the majority of cases [1]. However, a subgroup of patients does not respond to outpatient treatment or cannot be managed safely or practicably on an outpatient basis. In these cases inpatient or day patient treatment is needed [1]. Most of these patients have anorexia nervosa.

Inpatient treatment for anorexia nervosa produces a faster weight gain than outpatient treatment [2] and is often successful in the short term in that weight is generally restored to a healthy level. The problem is that few patients are able to maintain their new higher weight: instead, a large proportion relapse [3,4,5,6,7] and as a result during the first year following discharge 30-50% of patients need to be rehospitalized [8,9,10]. This failure to maintain the changes achieved in hospital has led to interest in developing post-hospitalization means of preventing the deterioration that follows discharge. An initial report suggested that fluoxetine might have this effect [11], but this was not confirmed in a subsequent controlled trial [7]. There is preliminary evidence that a form of cognitive behaviour therapy (CBT) might be beneficial [12,13], but this remains to be substantiated.

In the present study we adopted a different strategy. This was to modify the inpatient aspect of treatment, the goal being to reduce patients' propensity to relapse on discharge. To this end, the usual eclectic approach to inpatient treatment was replaced with a programme based on ‘enhanced’ cognitive behaviour therapy for eating disorders (CBT-E) [14] as this form of treatment is explicitly designed to produce enduring change. To this end, it focuses both on modifying the mechanisms thought to perpetuate eating disorder psychopathology [15] and on developing personalized relapse prevention skills [16]. The treatment has been found in two independent studies (combined n = 245) to produce sustained change in those eating disorder patients who are not significantly underweight, that is, those with bulimia nervosa or eating disorder not otherwise specified [17,18]. It has also been shown to be associated with a well-maintained and good outcome in two cohorts of adults with anorexia nervosa (total n = 99) [19] as well as a cohort of adolescents (n = 46) [20].

There are two forms of CBT-E, a focused form (CBT-Ef) that targets eating disorder psychopathology exclusively, and a more complex broad form (CBT-Eb) that also addresses certain additional problems (i.e., mood intolerance, clinical perfectionism, low self-esteem, and interpersonal difficulties) that in some patients appear to maintain the eating disorder psychopathology [15,21]. An outpatient study of CBT-E with patients who were not underweight (i.e., those with bulimia nervosa or eating disorder not otherwise specified) found that in those with substantial additional psychopathology of the type targeted in CBT-Eb, this version of was more effective than the focused form, whereas in the remaining patients the opposite was the case [18], although overall the two treatments were equally effective. To date, no data are available on the relative effects of these two forms of CBT-E in the treatment of patients with anorexia nervosa.

The present study was designed to address three key clinical questions and to compare the effects of the two CBT-E inpatient programmes. The three questions were: (1) Among patients with marked anorexia nervosa, what proportion is able to complete inpatient CBT-E? (2) Among those patients who can complete the treatment, what is their outcome? (3) Are the changes sustained?

## Method

### Design

A randomized controlled trial was conducted at an eating disorder inpatient unit. Eligible patients were randomly assigned to the two programmes, inpatient CBT-Ef or inpatient CBT-Eb. Patients were assessed before treatment, after the end of treatment, and 6 and 12 months later. The period of hospitalization lasted 20 weeks, the first 13 weeks being on an inpatient basis and the remaining 7 being as a day patient. The ethics committee of the Local Health Unit 22-Bussolengo approved the study (Study Protocol No. 86496 USL22, approved 20/12/05), and all participants (or their legal guardians for patients under 18) gave written informed consent to participation and to the anonymous use of personal data.

### Recruitment

**Fig. 1:**
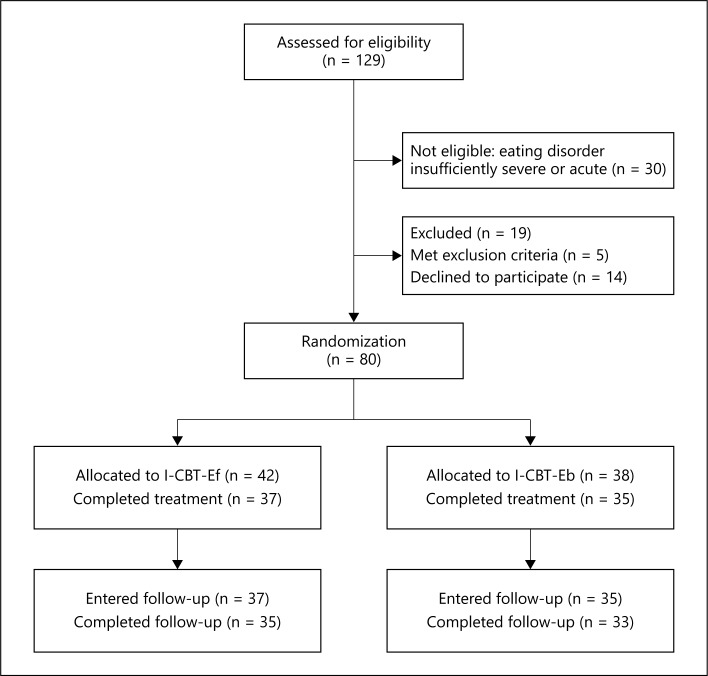
CONSORT flow diagram.

The sample was recruited from consecutive referrals to the eating disorder inpatient unit of Villa Garda Hospital (Northern Italy). The source of the referrals was heterogeneous. It included family doctors and secondary care health professionals (i.e., eating disorder specialists, outpatient eating disorder units of the National Italian Health System, general psychiatrists, and acute internal medicine units). Patients had to be aged between 14 and 65 years, to fulfil the DSM-IV diagnostic criteria for anorexia nervosa [22] as judged both by the referring clinician and by an eating disorder specialist (R.D.G.), and to require inpatient treatment either as a result of failure of outpatient treatment or because the eating disorder could not be managed safely on an outpatient basis. Eighty-one percent (80/99) of the eligible patients accepted the treatment, and were added to the unit's waiting list of up to 8 weeks. During the period on the waiting list the patients were managed by the referring agency. Five percent (5/99) of the eligible patients were excluded for the following reasons (an acute psychotic state, n = 2; significant substance abuse, n = 3), while 14% (14/99) declined to participate. Figure 1 shows the flow of participants through the study.

### Intervention

The two inpatient CBT-E programmes were derived from outpatient-based CBT-E. Both were designed to ensure a unified, rather than eclectic, approach to the patient's inpatient treatment [14]. The programmes retained all the main strategies and procedures of CBT-E. These were provided in both individual CBT-E sessions and in a group format. However, the programmes differed from outpatient CBT-E in that there was assistance with eating in the early weeks of hospitalization. This was provided by dietitians. Details of the programmes are provided elsewhere [23,24,25].

CBT-E includes many strategies and procedures designed to ensure that the changes made during treatment are well maintained [16]. The two inpatient programmes included three additional elements designed to reduce the high rate of relapse that typically follows discharge from hospital. First, the inpatient unit was ‘open’ with patients being free to go outside. In this way they continued to be exposed to the types of environmental stimuli that tend to provoke the return of eating disorder psychopathology. Second, during the 6 weeks prior to discharge, effort was made on a case-by-case basis to identify their likely triggers of setbacks, that they might have been sheltered from when in hospital. These were then addressed in the individual CBT-E sessions. Third, towards the end of treatment significant others were helped to create a positive stress-free home environment in readiness for the patient's return.

As in outpatient CBT-E, the first 4 weeks of the two programmes were identical and solely addressed the eating disorder psychopathology. Thereafter they diverged. In CBT-Ef the remaining sessions were focused on the eating disorder features (e.g., completing weight restoration, the overevaluation with shape and weight, dietary restraint, binge eating, and purging) [16], whereas in CBT-Eb the sessions also addressed mood intolerance, clinical perfectionism, low self-esteem, or interpersonal difficulties, as indicated in the individual patient [21].

Four clinical psychologists provided the individual CBT-E sessions. All had generic clinical experience and experience treating patients with eating disorder. Each conducted both treatments. Weekly supervision meetings with the psychologists and the inpatient team were led by R.D.G. and twice a year by C.G.F. All the individual sessions were recorded and were regularly audited to ensure that both treatments were well implemented. A substitute therapist stepped in when the primary CBT-E therapist had to be absent.

Psychotropic medication was not prescribed during the treatment, and during the first 2 weeks of hospitalization the psychotropic drugs taken at admission by patients were gradually phased out.

### Assessment

*Body Weight and Body Mass Index.* Weight was measured using a beam balance scale and height was measured using a wall-mounted stadiometer. Participants were weighed wearing only underwear.

*Eating Disorder Features.* These were assessed using the validated Italian version of the 12th edition of the Eating Disorder Examination (EDE) interview [26,27]. The EDE was administered by assessors who were trained and supervised by R.D.G., an expert on the instrument. The assessors were blind to the patients' treatment condition and had no involvement with treatment.

*General Psychiatric Features.* These were measured using the validated Italian version of the Brief Symptom Inventory [28,29], a short version of the Symptom Checklist-90 [30]. The Structured Clinical Interview for DSM-IV [31] was used at baseline to identify the presence of coexisting axis I psychiatric disorders.

### Power and Sample Size

Sample size calculations were performed a priori on an intent-to-treat basis. It was calculated that a sample size of 41 patients per treatment programme was required to provide 80% power at two-sided p < 0.05 to detect a difference in global EDE change of 0.45 points, assuming a standard deviation (SD) of global EDE change scores of 1.0 (14) (i.e., a moderate effect size of 0.45), and to detect a difference between the two programmes of at least 25% in the categorical outcome measure.

### Randomization

A computer-based minimization algorithm was used by one of the authors (H.D., who had no involvement in recruitment) to allocate patients to the two programmes, balancing age, gender, eating disorder diagnosis, and body mass index (BMI). When groups were evenly balanced, pre-prepared blocked randomization lists of varying size were used to allocate patients to the two treatments.

### Statistical Methods

The statistical analysis was undertaken by S.C. using standard treatment research data analytic procedures. Data are presented as numbers (%) for categorical data and as means (with SD) or medians (with range) for continuous data. Differences between groups were expressed as difference in proportion for categorical data and as mean difference for continuous data; χ^2^ or Fisher's exact tests (as appropriate) were used to compare categorical measures between the two groups, and t tests or Mann-Whitney tests (as appropriate for the distribution of the data) to compare continuous measures.

Change scores were calculated. For data assessed at any one time point, categorical data were compared using χ^2^ tests. Continuous data were compared using grouped t tests. Follow-up data were analysed using Cochrane Q test or Kendall test for categorical data, as appropriate, and repeated-measures analysis of variance for continuous data, to take into account the correlation between repeated measurements and to examine main effects and their interaction. Unless otherwise stated, the analyses were by intent-to-treat with the initial data brought forward. Other imputation methods were tested, but as there were few missing data, this made little difference to the main findings.

All statistical analyses were carried out by SPSS version 20.0 (SPSS Inc., Chicago, Ill., USA).

## Results

Sample

A total of 80 eligible patients were recruited and randomized to the two programmes (CBT-Ef, n = 42, or inpatient CBT-Eb, n = 38, fig. 1). The characteristics and clinical features of the sample and of the patients randomized to CBT-Ef and CBT-Eb are shown in table 1. The great majority were female (78/80; 97.5%) and most were in their twenties (34/80; 42.5%). Twenty-three patients (28.8%) were younger than 18 years. The patients were extremely underweight: 78.8% (63/80) had a BMI lower than 16.0. The median duration of anorexia nervosa was 5 years and 90% (72/80) had received prior treatment for the eating disorder. There were no significant differences between the two samples on the demographic and clinical variables.

### Intent-to-Treat Findings at End of Treatment and at 6 and 12 Months after Discharge

The primary goal of this study was to determine the proportion of patients with severe anorexia nervosa that could complete a CBT-Ef inpatient programme, and their treatment response. Intent-to-treat data are reported in table 2. The method of data imputation involved moving the last available data point forward as this has been the most commonly used approach in the studies to date. By the end of treatment the mean intent-to-treat BMI had increased from 14.3 (SD 1.7) to 18.9 (SD 1.5) in the whole sample, with there being no differences between the two CBT-E programmes. The mean BMI decreased to 17.8 (SD 2.2) at 6 months with there being no significant differences between the programmes. This decline stabilized after 6 months. In contrast, the improvement in eating disorder psychopathology and general psychiatric features at the end of the treatment was maintained both at 6 and 12 months' follow-up in both programmes.

### Question 1: What Proportion of Patients Completes Inpatient CBT-E?

Ninety percent of the patients completed the two CBT-E inpatient programmes [CBT-Ef, n = 37, 88.1%; CBT-Eb, n = 35, 92.1%, χ^2^ (1, n = 72) = 0.36, p = 0.550]. Two patients did not complete the programmes for independent reasons (concomitant medical disease requiring admission to an acute medical unit) and 6 patients discharged themselves. There were no significant differences between the completers and dropouts with respect to their clinical status at baseline.

### Question 2: What Is the Outcome among Those Who Complete Inpatient CBT-E?

There was a substantial response to the two inpatient programmes among the completers with no significant differences between them (table 2). The mean weight gain was 12.7 kg (SD 4.6; 95% CI 11.7 to 13.8; p < 0.001), equivalent to a BMI increase of 4.8 (SD 1.7; 95% CI 4.4 to 5.2; p < 0.001). Over 85% (86.1%, 62/72) achieved a BMI ≥18.5 or the corresponding cut-off BMI percentile in patients under 18 years of age [32], with no differences between patients under or over 18 years [95.5% (21/22) vs. 82.0% (41/50), respectively, χ^2^ (1, n = 72) = 2.31, p = 0.128]. Eating disorder psychopathology and general psychiatric features also improved substantially with the mean global EDE score among treatment completers decreasing by 2.0 (SD 1.1; 95% CI 1.7 to 2.2; p < 0.001) and mean GSI decreasing by 1.0 (SD 0.8; 95% CI 0.9 to 1.2; p < 0.001). Almost 50% of the patients (48.6%, 35/72) had minimal residual eating disorder psychopathology, defined as having a global EDE score below 1 SD above the community mean [33] (i.e., <1.74), with no significant differences between adolescent (<18 years) and adult (≥18 years) patients.

### Question 3: Are the Changes Sustained following Inpatient CBT-E?

There was high compliance with the follow-up assessment protocol with 95.8% (n = 69) and 94.4% (n = 68) of the treatment completers being successfully reassessed at 6 and 12 months following discharge. As would be expected, about 90% of the patients (CBT-Ef, n = 30, 88.2%; CBT-Eb, n = 31, 91.2%) received some form of post-discharge treatment [χ^2^ (1, n = 68) = 0.16, p = 0.690). This varied in nature and intensity and was delivered by therapists living close to the patients' place of residence.

There were no significant differences between the two programmes with respect to BMI, eating disorder and general psychopathology over the period of follow-up (table 2). In the whole sample mean weight decreased from 50.1 kg at discharge to 47.1 kg and 46.9 kg at 6 and 12 months, respectively (t = 5.0, p < 0.001; t = 5.4, p < 0.001, respectively), and this was reflected in a decline in the proportion of treatment completers who had a BMI ≥18.5 – or the corresponding cut-offs BMI percentile in patients under 18 years of age [32]. This decline stabilized after 6 months (86.1% at discharge; 47.8% at 6 months; 50.0% at 12 months).

The proportion of patients with BMI ≥18.5 or the corresponding cut-off BMI percentile [32] was significantly higher among adolescent (<18 years) than adult (≥18 years) patients, both at 6- and 12-month follow-up [6-month follow-up: adolescents 70.0%, 14/20; adults 38.8%, 19/49; χ^2^ (1, n = 69) = 5.55, p = 0.018; 12-month follow-up: adolescents 81.0%, 17/21; adults 36.2%, 17/47; χ^2^ (1, n = 68) = 11.64, p = 0.001].

In contrast, there was no post-discharge return in eating disorder psychopathology or general psychiatric features: both remained stable over follow-up. As a result the proportion with minimal residual eating disorder psychopathology did not change from discharge [48.6% (35/72)] to 12-month follow-up [61.8% (42/68)] and similar results were found comparing the adolescent and adult patients.

### Additional Psychopathology and the Relative Effects of CBT-Ef and CBT-Eb

We performed an exploratory analysis to compare the outcome of patients with and without marked additional psychopathology of the type that CBT-Eb is designed to target (viz., mood intolerance, clinical perfectionism, low self-esteem, and interpersonal difficulties). The severity of this additional psychopathology was rated by each patient's CBT-E therapist following the first 4 weeks of treatment and before the predetermined allocation of the patient to one or other of the two forms of CBT-E. On this basis, and as in Fairburn et al. [18], we created two patient subgroups, one with marked additional psychopathology (at least two of the domains present) and one without. There were no differences between these two groups in their response to the two forms of CBT-E, either at the end of treatment or during follow-up.

## Discussion

The aim of this study was to compare the effects of two inpatient CBT-E treatment programmes and determine if they were enduring. One programme focused solely on eating disorder features and the other also addressed mood intolerance, clinical perfectionism, core low self-esteem or interpersonal difficulties. To this end, a sizeable cohort of patients with anorexia nervosa was treated and then reassessed 6 and 12 months later. The sample was a severely affected one. Over 60% had a BMI lower than 15.0 and almost a quarter a BMI lower than 13.0.

There were four main findings. The first concerns the acceptability of inpatient CBT-E. Over 80% of the eligible patients agreed to embark upon the treatment and 90% completed it despite its explicit goal of full weight restoration.

The second finding is that the patients responded well to the two CBT-E programmes. Their mean weight gain was 12.7 kg with over three quarters gaining sufficient weight to enter the World Health Organization's healthy BMI range. In addition, almost 80% had minimal eating disorder psychopathology at discharge, despite the weight gain. This is of note as rapid weight gain and the accompanying change in shape activate eating disorder psychopathology in patients with anorexia nervosa.

The third finding concerns the patients' maintenance of change following discharge. This is of great importance as relapse is common after hospitalization. Here the findings are mixed. Whereas the marked improvements in eating disorder psychopathology and general psychiatric features achieved during treatment were sustained over follow-up, body weight fell somewhat during the first 6 months although it then stabilized in the whole sample. As one of the goals of CBT-E was to prevent deterioration of this type, this result is disappointing. This said, the fall in weight was modest, and it differed in its time course compared with that observed following other programmes [7]. In the present study the weight loss occurred exclusively during in the first 6 months whereas other studies have reported weight loss continuing over 9 [34] to 12 months following discharge [3,7]. In addition, the weight loss was limited mainly to adult patients, while the outcome of adolescent patients was excellent with 17 of 23 patients (73.9%) having a BMI percentile corresponding to the 18.5 cut-off [32] at 12-month follow-up. These data are consistent with the impression that treatment outcome among adolescents with anorexia nervosa is generally better than that among adults [35].

The fourth finding was that there were no differences at any point between the two inpatient programmes. This mirrors the finding with outpatient CBT-E [18]. In the outpatient study, however, it was found that the broad version of CBT-E was better suited to the treatment of those patients with marked difficulties of the type that it targeted whereas this was not found in the present study. Four explanations may be proposed to account for the similar overall outcome of the two inpatient programmes. First, in patients who are severely underweight it is possible that the eating disorder psychopathology is less influenced by external maintaining mechanisms of the type targeted by the broad version of CBT-E. Alternatively, these mechanisms may not be active when patients are in hospital as, for example, in the case of clinical perfectionism in the domain of school performance. Third, the individual CBT-E sessions were added to a complex programme that included many other elements and these may have overwhelmed any distinctive effects of the two forms of CBT-E. The fourth explanation is that CBT-Eb was not well implemented and as a result differed little from the focused form. This explanation is unlikely given the amount of training and supervision that the therapists were provided. Whatever the explanation, for the moment it seems that there is no benefit from using the more complex broad form of CBT-E with these patients.

The study had certain strengths. First, the first three findings are likely to be both robust and generalizable. This is because the cohort was sizeable and representative as it was recruited from an inpatient unit of the National Italian Health System. Second, the cases were not mild. All the patients were in the anorexia nervosa weight range and over three quarters had a BMI below 16.0. Third, CBT-E was these patients' sole psychological treatment: no other interventions were taking place in the background, both during the individual sessions and in the other elements of the programme. Fourth, the patients were followed up for over a year, the period when relapse is most likely to occur. Fifth, all clinical markers were examined using both psychometric validation procedures and clinical judgement (micro- and macro-analysis) [36].

The main limitations of the study were as follows. First, and inevitably, the period of follow-up was not closed: almost all the patients received subsequent outpatient treatment thereby complicating the interpretation of the follow-up findings. Second, a more extended period of follow-up would have been desirable to see whether the treatment effects persisted in the longer term. Third, no measures of mood intolerance, clinical perfectionism, core low self-esteem or interpersonal difficulties were employed thereby precluding moderator analyses of the type used in the equivalent outpatient study [18]. Fourth, the study did not include a comparison group treated with other forms of inpatient treatment. This was for logistical reasons as it is extremely difficult comparing different inpatient programmes. This limitation means that it is not possible to claim that the outcome obtained with these two forms of inpatient CBT-E were any different from those that would have been obtained using other approaches. Fifth, the results may not be generalizable to other inpatient settings. Sixth, given the design of the study, we cannot conclude that the changes observed were attributable to the CBT-E components of the two programmes rather than shared non-CBT-E elements.

In conclusion, this study has demonstrated that inpatient CBT-E is well accepted by patients with severe anorexia nervosa and the response is promising. Ninety percent completed the programme and most improved substantially. Deterioration after discharge did occur but it was not marked and it was for a limited period of time. While it cannot be claimed that inpatient CBT-E is superior to other forms of inpatient treatment, inpatient CBT-E has an important strength. This is that it is fully compatible with outpatient-based CBT-E, a treatment well suited to these patients' post-hospitalization care [17,18,19,20]. Thus there is potential for patients to move ‘seamlessly’ from inpatient care to day patient care, and then on to outpatient treatment with no change in their form of treatment. This harmonization of approaches might improve these patients' longer-term outcome as it would avoid the changes in therapeutic approach that often accompany such transitions.

## Figures and Tables

**Table 1:** Characteristics of the two samples at baseline

Characteristics	All patients (n = 80)	CBT-Ef (n = 42)	CBT-Eb (n = 38)	Test	^p^ value
Age, years	23.4 (6.9)	23.1 (6.8)	23.7 (7.0)	–0.41	0.681
Gender, n (%) female	78 (97.5)	40 (95.2)	38 (100)	1.86	0.173
Marital status, n (%)					
Single, never married	72 (90.0)	39 (92.9)	33 (86.8)	0.97	0.616
Married or living as such	6 (7.5)	2 (4.8)	4 (10.5)		
Separated or divorced	2 (2.5)	1 (1.3)	1 (1.3)		
Occupation, n (%)				2.75	0.600
Higher	1 (1.3)	0 (0)	1 (2.7)		
Intermediate	17 (21.3)	8 (19.0)	9 (24.3)		
Lower	4 (5.0)	3 (7.1)	1 (2.7)		
Unclassifiable	19 (23.8)	9 (21.4)	10 (27.0)		
Full-time student	38 (47.5)	22 (52.4)	16 (43.2)		
Median duration of eating disorder (range), years	5.0 (0–26.0)	4 (0–20.0)	5.0 (0–26.0)	–0.65	0.517
Current other axis I disorder^a^					
Major depressive episode, n (%) present	45 (56.3)	25 (59.5)	20 (52.6)	0.38	0.535
Any anxiety disorder, n (%) present	16 (20.0)	9 (21.4)	7 (18.4)	0.11	0.737

Data are shown as mean (SD) unless otherwise indicated.

a The Structured Clinical Interview for DSM-IV was used to assess depression and anxiety disorders.

**Table 2:** Characteristics of the two samples before treatment, after treatment, at 6- and 12-month follow-up among those who completed treatment

	Before treatment			After treatment			6-month follow-up			12-month follow-up		
	CBT-Ef (n = 37)	CBT-Eb (n = 35)	total (n = 72)	CBT-Ef (n = 37)	CBT-Eb (n = 35)	total (n = 72)	CBT-Ef (n = 36)	CBT-Eb (n = 33)	total (n = 69)	CBT-Ef (n = 34)	CBT-Eb (n = 34)	total (n = 68)
Body weight, kg	37.4 (5.6)	37.3 (5.3)	37.4 (5.4)	50.0 (5.8)	50.3 (4.4)	50.1 (5.1)^a^	45.8 (6.7)	48.0 (7.7)	46.8 (7.2)^a^, ^b^	46.5 (6.9)	47.0 (7.1)	46.7 (7.0)^a^, ^b^
BMI	14.3 (1.8)	14.3 (1.8)	14.3 (1.8)	19.2 (1.3)	19.3 (0.7)	19.2 (1.3)^a^	17.6 (2.5)	18.3 (1.9)	17.9 (2.3)^a^, ^b^	17.9 (2.4)	17.8 (2.2)	17.8 (2.3)^a^, ^b^
BMI ≥ 18.5, n (%)	0	0	0	32 (86.6)	29 (82.9)	62 (86.1)	16 (44.4)	17 (51.5)	33 (47.8)	18 (52.9)	14 (41.2)	34 (50.0)
Eating disorder psychopathology												
Overall severity (global EDE)	3.9 (1.1)	3.4(1.1)	3.7 (1.1)	1.7 (1.1)	1.7 (1.1)	1.7 (1.0)^a^	2.0 (1.5)	1.4 (1.2)	1.7 (1.4)^a^	1.6 (1.5)	1.6 (1.2)	1.6 (1.3)^a^
Global EDE less than 1 SD above community mean, n (%)	1 (2.7)	2 (5.7)	3 (3.8)	20 (54.1)	17 (48.6)	37 (51.4)	21 (58.3)	21 (63.6)	42 (60.9)	20 (54.1)	22 (62.9)	42 (61.8)
Dietary restraint (EDE subscale)	4.3 (1.3)	3.8 (1.1)	4.1 (1.2)	0.8 (0.8)	0.8 (0.7)	0.8 (0.8)^a^	1.7 (1.9)	1.1 (1.4)	1.4 (1.7)^a^, ^b^	1.4 (1.8)	1.5 (1.5)	1.5 (1.7)^a^, ^b^
Eating concern (EDE subscale)	3.5 (1.3)	3.2 (1.4)	3.4 (1.3)	1.2 (1.1)	1.1 (1.0)	1.2 (1.1)^a^	1.8 (1.5)	1.4 (1.4)	1.6 (1.5)^a^	1.4 (1.5)	1.4(1.3)	1.4 (1.4)^a^
Weight concern (EDE subscale)	3.9 (1.6)	3.3 (1.6)	3.6 (1.6)	2.0 (1.3)	2.1 (1.4)	2.1 (1.4)^a^	1.9 (1.6)	1.4 (1.2)	1.7 (1.4)^a^	1.7 (1.6)	1.4(1.3)	1.5 (1.4)^a^, ^b^
Shape concern (EDE subscale)	4.0 (1.4)	3.3 (1.6)	3.7 (1.5)	2.8 (1.6)	2.7 (1.6)	2.8 (1.6)^a^	2.5 (1.7)	1.8 (1.3)	2.2 (1.6)^a^, ^b^	2.0 (1.8)	2.0 (1.3)	2.2 (1.6)^a^, ^b^
EDE												
Objective bulimic episodes, n (%) present	11 (29.7)	12 (34.3)	23 (31.9)	1 (2.7)	3 (8.6)	4 (5.6)	5 (13.9)	5 (15.2)	10 (14.5)	3 (8.1)	5 (14.3)	8 (11.8)
If present, episodes/28 days, median (range)	10.0 (1–148)	6.0 (1–126)	8.0 (1–148)	9.0 –	2.0 (1–9)	5.5 (1–9)	15.0 (1–30)	6.0 (1–15)	8.0 (1–30)	1.0 (1–6)	15.0 (1–30)	8.0 (1–30)
Self-induced vomiting, n (%) present	12 (32.4)	13 (37.1)	25 (34.7)	6 (16.2)	3 (8.6)	9 (12.5)	8 (22.2)	5 (15.2)	13 (18.8)	6 (16.2)	8 (22.9)	14 (20.6)
If present, episodes/28 days, median (range)	21.5 (8–148)	30.0 (3–140)	23.0 (3–148)	2.0 (1–9)	1.0 (1–2)	2.0 (1–9)	12.5 (2–40)	5.0 (1–178)	5 (1–178)	7.5 (1–28)	10.0 (2–70)	9.0 (1–70)
Laxative misuse, n (%) present	9 (24.3)	10 (28.6)	19 (26.4)	0	0	0	1 (2.8)	2 (6.1)	3 (4.3)	1 (2.7)	3 (8.6)	4 (5.9)
If present, episodes/28 days, median (range)	20.0 (2–84)	28.0 (1–28)	28.0 (1–84)	–	–	–	6.0 –	14.5 (1–28)	6.0 (1–28)	9.0	15.0 (4–28)	12.0 (4–28)
General psychiatric features, GSI	1.9 (0.8)	1.8 (0.8)	1.8 (0.8)	0.9 (0.7)	0.7 (0.6)	0.8 (0.6)^a^	1.2 (0.8)	0.9 (0.8)	1.0 (0.8)^a^	0.9 (0.7)	0.8 (0.7)	0.9 (0.7)^a^

Data are shown as mean (SD) unless otherwise stated. Global EDE was less than 1 SD above community EDE mean for young adult women (i.e., below 1.74). GSI = Global Severity Index. No differences were found between CBT-Ef and CBT-Eb at each time point. on total sample:

a p < 0.05 vs. before treatment;

b p < 0.05 vs. end of therapy.
